# Spontaneous tumor regression and immunotherapy response demonstrate clonal T-cell expansion in Merkel cell carcinoma

**DOI:** 10.1038/s41698-025-00987-6

**Published:** 2025-07-01

**Authors:** Patrick Hallaert, John W. Roman, Mairead Baker, Natasha T. Hill, Jennifer L. Marte, James L. Gulley, Nicholas Logemann, Courtney W. Hudgens, Alexandre Reuben, Isaac Brownell

**Affiliations:** 1 Dermatology Branch, National Institute of Arthritis and Musculoskeletal and Skin Diseases (NIAMS), National Institutes of Health (NIH), Bethesda, MD USA; 2 Department of Dermatology, Naval Medical Center Portsmouth (NMCP), Portsmouth, VA USA; 3 Center for Immuno-Oncology, Center for Cancer Research (CCR), National Cancer Institute (NCI), National Institutes of Health (NIH), Bethesda, MD USA; 4 Department of Dermatology, Walter Reed National Military Medical Center (WRNMMC), Bethesda, MD USA; 5 Department of Translational Molecular Pathology, The University of Texas MD Anderson Cancer Center, Houston, TX USA; 6 Department of Thoracic/Head & Neck Medical Oncology, The University of Texas MD Anderson Cancer Center, Houston, TX USA

**Keywords:** Oncology, Skin cancer, Tumour immunology

## Abstract

Merkel cell carcinoma (MCC) is a rare and aggressive neuroendocrine skin cancer that is responsive to immune checkpoint inhibitors (ICI). On rare occasion, MCC spontaneously regresses. It is speculated that this regression occurs when biopsy-induced antigen shedding precipitates an immune response. Here, we demonstrate the activation of an adaptive immune response in a patient whose tumor underwent spontaneous regression after biopsy. To evaluate the tumor immune microenvironment during regression, we performed quantitative immunohistochemical analysis and T-cell receptor (TCR) sequencing. Relative to baseline, the regressing tumor showed evidence of an activated cytotoxic T-cell response together with increased TCR clonality, greater representation of dominant T-cell clones, and the emergence of novel high-frequency T-cell clones. Similar changes in TCR profiles were observed in an MCC tumor undergoing ICI-induced regression. Taken together, our results provide evidence that the expansion of novel and pre-existing adaptive immune responses drives spontaneous MCC regression.

## Introduction

Spontaneous tumor regression (STR) occurs when tumors involute without treatment. Although uncommon, this phenomenon has been reported in a wide range of malignancies^1^. Since STR most often takes place shortly after a diagnostic biopsy, it is hypothesized that local trauma may release tumor antigens, precipitating an anti-tumor immune response that drives regression^1–3^.

Merkel cell carcinoma (MCC) is a rare and aggressive neuroendocrine skin cancer that has a high response rate to immunotherapy^4^. MCC can develop either through the integration of the Merkel cell polyomavirus (MCPyV) into the host genome or through the progressive acquisition of UV-induced somatic mutations^5^. Despite having an incidence of only 0.7 per 100,000 person-years, to date over 50 cases of MCC STR have been reported in the literature^2,6,7^. These existing studies have primarily employed immunohistochemistry to identify cytotoxic T-cells in STR-associated immune infiltrate^2,3,8^. However, definitive evidence of the immune mechanisms governing STR in MCC remains evasive.

Here, we explore the immune response underlying spontaneous regression in an MCC tumor. Using quantitative immunohistochemistry (IHC), we characterized the intratumoral and peritumoral immune infiltrate present before and during spontaneous regression. We then harnessed T-cell receptor (TCR) sequencing to quantify adaptive T-cell responses during STR and found evidence of clonal expansion that was similar to an MCC tumor regressing after treatment with avelumab, an anti-PD-L1 immune checkpoint inhibitor (ICI)^9^.

## Results

To investigate the mechanism underlying spontaneous regression, we collected tumor samples from a patient experiencing STR. Tumor samples were obtained at two time points: first, at the time of biopsy, and second, as the tumor was regressing. As a comparator, we also analyzed tumor samples from a patient whose MCC tumor underwent regression after the use of the ICI avelumab.

### STR Patient

**Fig. 1:**
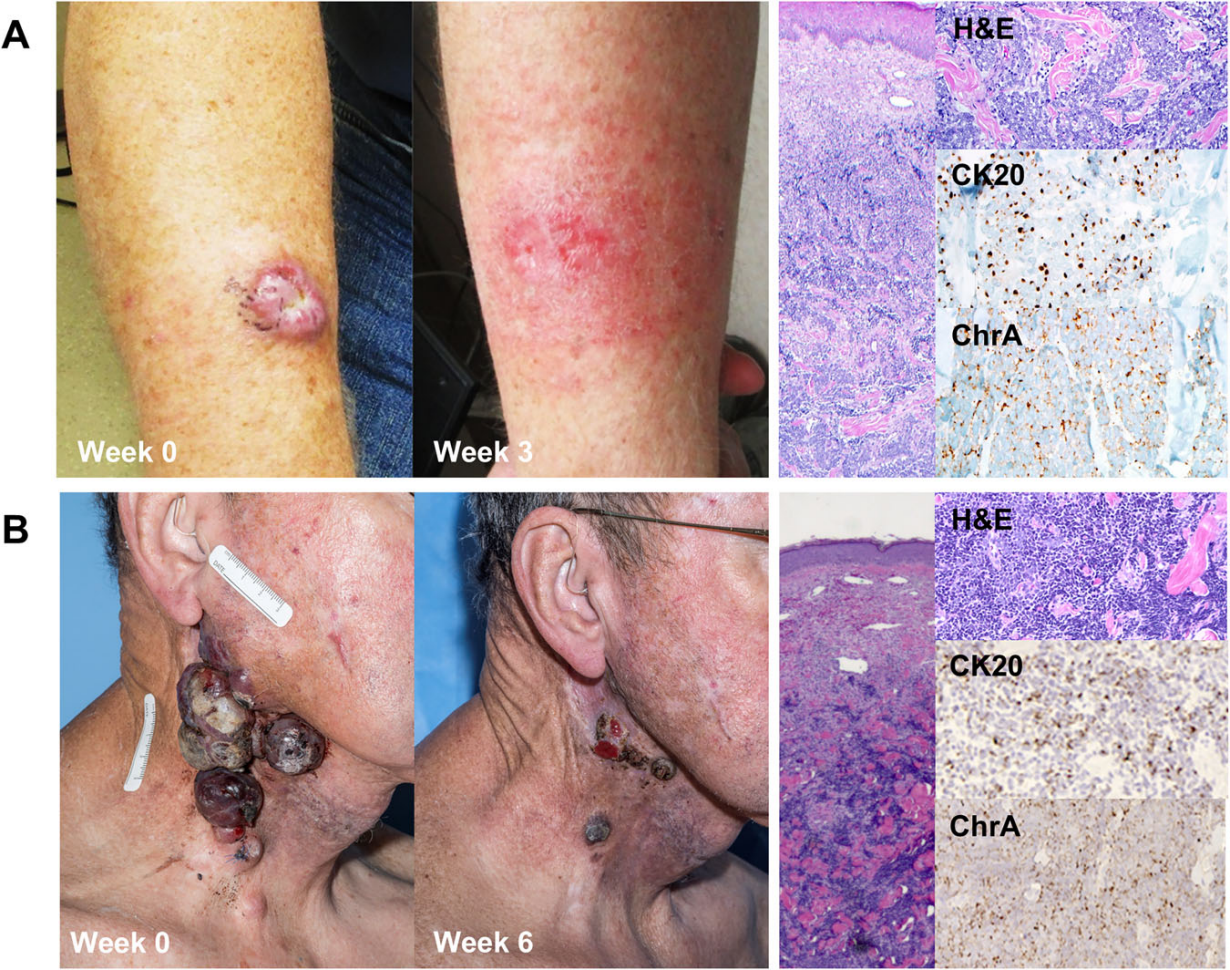
Clinical and histological appearance of Merkel cell carcinoma in a case of spontaneous tumor regression and avelumab-driven regression. **A** Tumor at baseline and during spontaneous regression at week 3 (left), with corresponding Hematoxylin and Eosin (H&E), Keratin 20 (CK20), and Chromogranin A (ChrA) staining at baseline (right). **B** Tumor at baseline and during avelumab-mediated regression at week 6 (left), with corresponding H&E, CK20, and ChrA staining at baseline (right). Original magnifications of micrographs 100x and 200x.

**Fig. 2:**
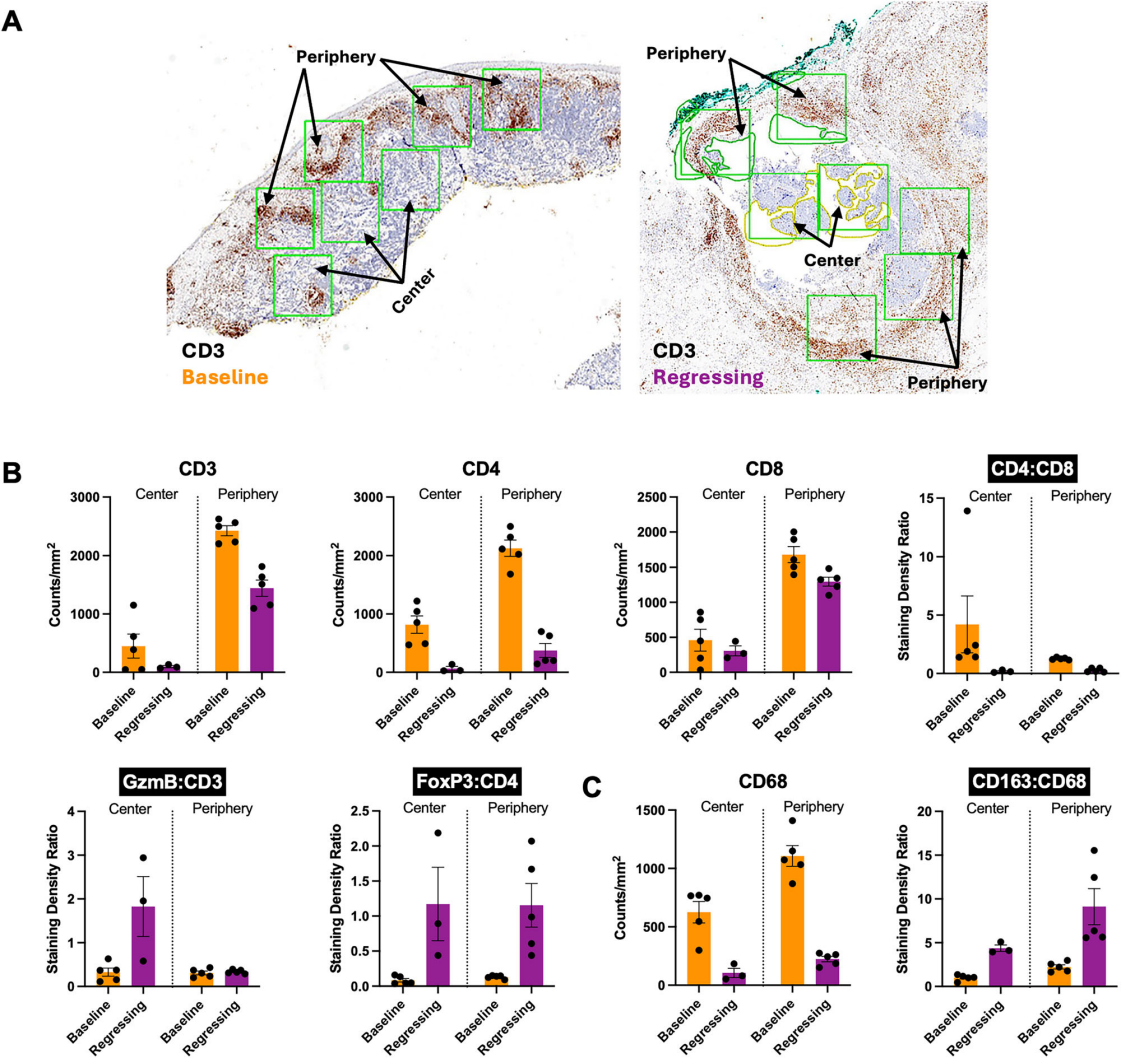
Intratumoral and peritumoral immune cell densities and density ratios at baseline and during spontaneous MCC regression. **A** Immunohistochemical (IHC) staining for CD3 (brown) with hematoxylin counterstain in tissue sections at baseline and during spontaneous tumor regression (regressing). Intratumoral (center) and peritumoral (periphery) CD3 densities were quantified by counting stained cells in 1 mm^2^ regions (green squares). This method was also used to determine the densities of other immune cells identified by IHC staining. **B** Quantitative IHC analysis of T-cell markers CD3, CD4, and CD8 as well as CD4:CD8, GzmB:CD3, and FoxP3:CD4 density ratios. **C** Quantitative IHC analysis of macrophage marker CD68 as well as CD163:CD68 density ratio.

A 65-year-old man presented with a twelve-week history of a rapidly growing pink 3.5 cm skin tumor on his right forearm (Fig. 1A). A partial shave biopsy was obtained. Histopathological and immunohistochemical findings, as well as MCPyV PCR, diagnosed the lesion as polyomavirus-positive MCC. Eight days after biopsy, the remaining tumor spontaneously regressed with rapid shrinking of the clinical lesion. Histopathological analysis of a wide local excision specimen obtained 45 days after the biopsy revealed residual focal dermal nests of small blue tumor cells and a surrounding lymphohistiocytic inflammatory infiltrate (Fig. 2A). There was marked apoptosis of tumor cells and broad areas of necrosis and hemorrhage, consistent with a regressing tumor.

### ICI Patient

An 82-year-old man presented with metastatic polyomavirus-negative MCC of the right neck (Fig. 1B). Prior treatment included wide local excision and carboplatin plus etoposide. Six months after completing treatment, recurrence was diagnosed by positron emission tomography (PET). Consequently, the patient was started on avelumab, a PD-L1 inhibitor^9^. The patient’s skin tumor was biopsied prior to initiating therapy as well as during tumor regression 7 weeks after starting treatment. The initial biopsy was consistent with MCC, while the biopsy at 7 weeks showed only a dense lymphohistiocytic infiltrate with no residual tumor.

### STR-associated changes in immune infiltrate suggest an activated cellular immune response

We employed immunostaining for CD3, CD4, CD8, Granzyme B (GzmB), FOXP3, CD163, and CD68 markers to quantitively analyze the STR patient’s tumor immune landscape both at baseline and during STR (Fig. 2)^10–12^. Comparisons were also made between the intratumoral immunocytes at the center of the tumor versus the peritumoral infiltrates at the tumor periphery.

The baseline tumor demonstrated an acute lymphohistiocytic immune infiltrate that was denser at the tumor periphery than at the center. The regressing tumor showed similar intratumoral and peritumoral infiltrates which had a lower cell density compared to baseline. Consistent with this, the regressing tumor exhibited a reduction in cell staining for lymphocyte markers such as CD3, CD4, and CD8 as well as the monocyte/macrophage marker CD68 (Fig. 2).

Although there were fewer immunocytes in the regressing tumor, there was also a decrease in the CD4:CD8 ratio and an increase in the intratumoral GzmB:CD3 ratio, suggesting that STR was driven by cytotoxic CD8+ T-cells. Furthermore, the regressing tumor exhibited a rise in both CD163:CD68 and FoxP3:CD4 ratios, suggesting that the inflammation driving STR was associated with an upregulation in immunosuppressive M2 macrophages and regulatory T-cells (Tregs), respectively (Fig. 2). Such immunoregulatory cells are expected to be recruited to sites of persistent cytotoxic CD8+ inflammation^13^. A similar immunohistochemical analysis of the tumor regressing in response to ICI was not possible as there was no residual tumor present in the regressing tumor biopsy.

### STR is associated with the expansion of TCR clones

**Fig. 3:**
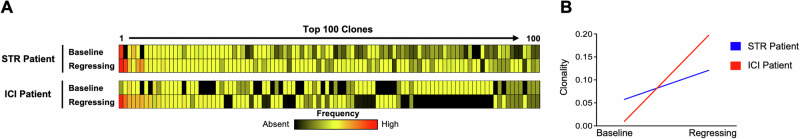
MCC regression is associated with increased TCR clonality, heightened representation of dominant clones, and the emergence of new high-frequency clones. **A** Map of the 100 most dominant individual T-cell clones, averaged across paired baseline and regressing tumor samples, organized from most to least frequently represented in tumors with spontaneous tumor regression (“STR Patient”) and regression after avelumab treatment (“ICI Patient”). **B** Both tumors displayed an increase in T-cell receptor clonality during regression when compared to their baseline.

TCR sequencing was used to characterize the dominant T cell clones present at baseline and during MCC STR. Analysis of the resulting top 100 TCR clones indicated that regression was associated with an increase in TCR clonality, an upregulation in dominant T-cell clones, and the emergence of novel high-frequency clones (Fig. 3). TCR sequencing in the MCC tumor undergoing ICI-mediated tumor regression showed a similar increase in T-cell clonality that also involved the expansion of both novel and pre-existing clones.

## Discussion

Our results strongly suggest that spontaneous regression is driven by an adaptive cytotoxic T-cell response targeting tumor antigens. This process is accompanied by the expansion of discrete TCR clones as well as an upregulation of immunosuppressive effector cells. In addition, our TCR sequencing analysis suggests that this process bears similarity to the adaptive T-cell response governing ICI-mediated tumor regression.

Since spontaneous regression is uncommon and unpredictable, few studies have scrutinized the immunological basis of STR, especially in a cancer as rare as MCC. Initial reports of spontaneously regressing MCCs described the migration of lymphocytes into and around the tumor^2,14,15^. Notably, when comparing four spontaneously regressing MCCs to three non-regressing MCCs, Inoue et al. found that tumor-infiltrating lymphocytes (TILs) were associated with spontaneous regression^16^. This link was further substantiated by Terui et al., who found increased IHC staining for CD8+ T-cells at the time of biopsy in a tumor that subsequently underwent spontaneous regression when compared to five non-regressing tumors^8^. Other studies have similarly employed IHC to qualitatively assess immune infiltrates and have found dense infiltrates of intratumoral CD8+ T-cells and peripheral CD4+ T-cells at baseline in tumors that underwent regression^17,18^. The immune infiltration associated with MCC tumors mid-regression is less well explored.

To elaborate on prior immunohistochemical descriptions of STR, we harnessed automated image analysis to objectively map immune cell densities in a spontaneously regressing MCC tumor. Building on previous studies, our results provide evidence for a cytotoxic T-cell driven immune response during regression. Intriguingly, we also found that STR was associated with an expansion in the immunosuppressive cell repertoire. The observed upregulation of Tregs and M2 macrophages could function to restore immune homeostasis and control excessive tissue damage after a robust anti-tumor inflammatory response^13^.

We also examined the distribution of immune cells both at baseline and during STR. This analysis was prompted by our prior finding that in cases of MCC where the density of peritumoral CD8+ and CD3+ T-cell were above a median value (791.1 and 1811.4 cells/mm^2^ respectively), there was improved overall survival^10^. Interestingly, the STR patient’s tumor displayed average peripheral CD8+ and CD3+ densities of 1609.9 and 2497.2 cells/mm^2^ respectively at baseline, which are notably higher than our previously reported medians. This is consistent with the fact that spontaneous regression in MCC typically confers a favorable prognosis^19^. Indeed, at the time of writing, this patient is alive with no evidence of recurrent MCC, 7 years after he first experienced STR.

We also observed that MCC STR was accompanied by increased TCR clonality, the emergence of novel high-frequency clones, and the expansion of pre-existing high-frequency clones. In the past, research has similarly found increased T-cell clonality in cases of spontaneous regression in melanoma, although it has largely employed low-throughput clonality assays that less comprehensively examine changes in the TCR repertoire^20,21^. Our analysis of the MCC tumor treated with the ICI avelumab also detected similar changes in TCR clonality, which is consistent with previous research showing clonal expansions of TILs in patients with melanoma that are responsive to ICI treatment^22,23^.

In MCC specifically, most TCR sequencing has focused on detecting MCPyV-specific T-cells, and has found that the expansion of MCPyV-reactive T-cells and their infiltration into the tumor is linked to increased MCC survival^24,25^. In contrast to MCPyV xenoantigens, which provide immune targets in virus-positive MCC, virus-negative MCC (VN-MCC) express UV-induced neoantigens^26^. Interestingly, the clonal expansion of T-cells associated with a complete and durable response in the avelumab-treated VN-MCC tumor we analyzed suggests that VN-MCC neoantigens are also capable of driving adaptive anti-tumor immunity. This is consistent with a recent study that identified neoantigen-specific CD4+ T-cells in a patient with VN-MCC who similarly achieved a durable response to avelumab^26^.

Taken together, our study strongly suggests that STR in MCC is driven by adaptive polyclonal CD8+ T-cell immune responses that are analogous to those observed in ICI-mediated tumor regression. However, our study is limited by being a single case of STR, and by the fact that we did not have viable TILs or peripheral blood mononuclear cells (PBMCs) available for functional testing of tumor antigen recognition. Future studies using a larger sample size and functional immune assays will be needed to validate our observations, although this may pose a challenge given the rarity of both spontaneous regression and MCC. Additional research could also elucidate how biopsies trigger an immune response by identifying the target antigens of T-cell clones that drive tumor regression.

## Methods

### Immunohistochemistry

Tumor samples were fixed in formalin and embedded in paraffin. The resulting tissue blocks were cut into 4 μm sections and stained using CD3 (Polyclonal - Dako), CD4 (4B12 - Thermo Scientific), CD8 (C8/144B - Thermo Scientific), Granzyme B (11F1 - Cell Signaling Technologies), FoxP3 (Clone 206D - BioLegend), CD163 (10D6 - Leica Biosystems), and CD68 (PG-M1 - Dako) primary antibodies. Stained slides were then scanned using an Aperio Scanscope AT Turbo and staining was quantified digitally using Aperio ImageScope image analysis software as previously described^10–12^.

### T-cell receptor sequencing

TCR sequencing was performed using patient DNA that was extracted from formalin fixed, paraffin embedded (FFPE) tumor specimens collected at baseline or during tumor regression. As described previously^10,12^, the resulting CDR3 variable regions of T-cell receptor β-chains were then amplified and sequenced via ImmunoSEQ (Adaptive Biotechnologies, Seattle, WA).

### MCPyV detection PCR

Nested quantitative PCR (qPCR) was performed on 10 ng of tumor DNA extracted from paraffin-embedded sections using the QIAamp DNA FFPE tissue kit (Qiagen, cat. 56404). A two-step nested PCR amplification was used to quantify MCPyV DNA relative to human DNA at the thyroid peroxidase (*TPO*) locus. The first step used an outer MCPyV primer set: outer forward primer 5′ GGCAACATCCCTCTGATGAAAGC 3′ and outer reverse primer 5′ CCACCAGTCAAAACTTTCCCAAGTAGG 3′. In a separate reaction, the following *TPO* primers were used: TPO forward primer 5′ CCACACTGCCCATCTCGGAGAC 3′ and TPO reverse primer 5′ GCGGTGAGGTCCCTACGGCCTG 3′. A touchdown PCR strategy was used, starting with an annealing temperature of 64 °C and reducing by 3 °C per 5 cycles to reach 58 °C. 4% of the reaction volume was amplified in step two, a qPCR amplification with SYBR-Green detection using a StepOnePlus real time PCR cycler (Applied Biosystems). The MCPyV primers were: inner forward primer 5′ CTTAAAGCATCACCCTGATAAAGG 3′ and inner reverse primer 5′ AAACCAAAGAATAAAGCACTGATAGCA 3′. The same TPO primers from step one were also used in step two. MCPyV quantification relative to TPO was determined using delta cycle threshold (ΔCt) values, and a relative ratio was calculated. A threshold cutoff value for virus-positivity of 0.001 (1 copy MCPyV to 1000 copies TPO) was employed.

### Statistical analysis

Quantitative IHC plots were generated with GraphPad Prism v5.0 to show mean cell density values with the standard error of the mean. The ImmunoSEQ Analyzer software (Adaptive Biotechnologies, Seattle, WA) was employed to track the top 100 clones in each sample and to compute clonality.

## Data Availability

The data associated with this article will be shared upon reasonable request to the corresponding author.
